# Increase of Fever in Ireland

**Published:** 1847-07

**Authors:** 


*Increase of fever in Ireland.*—Deaths by famine are happily becoming rare, but fever is creating great ravages. The accounts from Kerry, Galway, Roscommon, and Longford, are of an extremely unfavourable character. In the union work-house of the latter county the number of deaths in the year ending the 1st of April, 1846, was 112, while for the corresponding period this year they amounted to 677.—*Ibid.*

